# Supplementary material for: Are carbon emissions trading and green financial instruments synergistic? -Comprehensive quantitative research based on content analysis
Source: PLoS One. 2024 Mar 7;19(3):e0298601. doi: 10.1371/journal.pone.0298601 (PMC10919601; doi:10.1371/journal.pone.0298601)
Supplement: S1 Data — (ZIP) [file pone.0298601.s001.zip › Data/地级市Z_F工作报告.zip!/║■▒▒/╗╞╕╘╩╨/└┤╫╘╣1⁄2╓┌║┼í╛┬φ┐╦╩2╛▌═°í┐_2016.docx]

各位代表：

　　现在，我代表县人民政府向大会报告工作，请连同《罗田县国民经济和社会发展第十三个五年规划纲要（草案）》一并审议，并请各位政协委员和其他列席人员提出意见。

　　一、2015年及“十二五”时期工作回顾

　　2015年，在市委、市政府和县委的正确领导下，在县人大和县政协的大力支持下，我们团结和依靠全县人民，主动适应新常态，全力以赴稳增长、促改革、调结构、惠民生。县域经济平稳发展。全年完成地区生产总值113.2亿元、增长8.8%，全社会固定资产投资171.5亿元、增长17.6%，社会消费品零售总额60.75亿元、增长10.5%，外贸出口5653万美元、增长15.9%，地方公共财政预算收入6.29亿元、增长16.95%。民生质量持续提升。城乡居民人均可支配收入分别为21027元和8493元、增长9.5%和9%，居民消费价格指数涨幅2%，城镇登记失业率2.5%。节能减排超额完成市定目标。社会治安满意度等“五率”指标居省市前列。干事创业成效显著。获批全国生态保护与建设示范区、全省首批碳排放交易项目试点县，成功举办2015中国有机农业大会，承办全省大别山革命老区扶贫开发振兴发展推进会和全市精准扶贫、城市建设管理等工作现场会。荣获全国森林旅游示范县、地质灾害防治高标准“十有县”[1]、首批规划建设管理示范县城和全省金融扶贫示范县、金融信用县、农村垃圾治理和电子商务进农村示范县。省级生态县和“六五”普法工作通过省级验收。《余三胜轶事》荣获中国第七届黄梅戏艺术节牡丹奖和湖北省“五个一”工程奖。土地卫片执法、科技成果引进转化、城市综合管理、移民后扶、减轻农民负担执法检查和涉农收费专项治理等工作在省市领先。

　　一年来，我们着力抓了七个方面工作：

　　（一）着力转型升级，经济质量不断提高

　　农业稳步发展。完成农业产值42亿元、增长6.9%，粮食生产实现“十二连增”。科技兴农和产业化经营水平不断提高，“两新”应用率90%，农机综合水平65%，培育龙头企业6家，新发展专业合作社161家，新增“三品一标”[2]产品7个，改造板栗低产林3万亩。休闲观光农业快速发展，新发展家庭农场21家。农业生产条件持续改善，实施土地整理项目9个、小（二）型水库除险加固工程21座，河西畈山洪沟治理工程被评为全省生态安全示范工程。惠农政策全面落实，发放直补资金5710万元。

　　工业逆势增长。完成规模工业增加值25.6亿元、增长7.9%，新增规模企业15家，经济运行质量居全市前列。园区建设全面提速，配套建设逐步完善，新开工项目25个，新建厂房20万㎡，建成投产项目9个，宏源药业整体退城入园。服务企业措施有力。完善入园企业服务管理办法，整治涉企“三乱”和企业周边环境；争取省调度资金和市大别山产业发展基金3.09亿元，调度县财政资金1.2亿元，担保融资11.3亿元，有效缓解企业资金困难。

　　旅游全面火爆。游客接待人数、旅游综合收入分别增长20.8%和22.8%。景区建设亮点纷呈。完成天堂寨景区观光电梯、玻璃栈道和薄刀峰景区游客服务中心、道路安防工程建设，三里畈大别山温泉度假区成功开园，燕儿谷等乡村休闲旅游景点建设有序推进。营销方式不断创新。与国内知名旅游网站和旅行社签订营销合作协议，智慧旅游、罗田文联、九资游等新平台、新媒体推介效果显著，游客接待人数呈现井喷式增长的良好势头。5A级景区和旅游标准化创建工作扎实推进，大别山风景名胜区创建通过评审，大别山世界地质公园申报以评审得分全国第一的成绩获得推荐。

　　（二）着力精准扶贫，脱贫措施不断完善

　　做好顶层设计。按照“六个精准”和“精准扶贫、不落一人”要求，制定“3355”实施意见和系列行动方案，并根据新的政策精神和工作实际，及时完善了“五个一批”[3]措施和资金整合等配套政策。

　　落实脱贫责任。建立和完善工作机制，层层签订“军令状”，全面完成精准识别工作。106个贫困村、45个难点村全部派驻第一书记和工作队，干部结亲帮扶贫困户实现全覆盖。

　　坚持示范带动。探索“五位一体”和“公司（合作社、支部、能人大户）+贫困户”等有效模式，着力推进黑山羊等五大产业精准扶贫工程，市场主体带动贫困户脱贫成效显著。易地扶贫搬迁工作走在省市前列，九资河五条路、胜利甘家冲、平湖东冲畈等首批示范点开工建设。整村推进基础设施建设工程全面启动。

　　（三）着力项目招商，发展后劲不断增强

　　项目建设成绩突出。争取国家项目223个，到位资金13.39亿元、同比净增2.4亿元，房管、交通、国土、供电、水利、教育等部门争取资金过亿元，移民、体育、扶贫、文化、环保等32个部门超额完成年度任务。项目建设投入加大。一方山水大酒店等5个全市“双百项目”完成投资4.9亿元。交通完成货币工程量11.47亿元，麻武高速罗田段建成通车，河铺连接线同步建设。扎实开展村村通客车“三万”活动，改造农村道路927公里、危桥32座，完成安保工程314公里，新建错车台2005个。建成园区、匡河2座变电站，改造供电线路639公里、配变台255个。新建移动通信基站125个。平坦原抽水蓄能、光伏发电、风力发电、沿江高铁罗田段等重大项目进展顺利。谋划“十三五”时期大项目417个、总投资2756亿元，一批项目挤进国家、省、市项目库。

　　招商引资成效显著。创新招商机制，出台支持返乡创业和小微企业、电商产业等系列文件，举行“招商周”，参加中国光彩事业老区行等系列活动，签订投资框架协议41个、协议投资167.6亿元。严把项目准入关，评审项目42个，正式签约项目31个、投资79.8亿元。纺织产业园、居然之家、华能光伏发电等项目成功落户。

　　（四）着力改革创新，发展环境不断优化

　　全面深化改革激发新活力。精简行政审批事项和审批资料，压缩办事时限，取消收费项目468项，部门办事效率和服务水平不断提高。实行“三证合一、一照一码”、“先照后证”[4]商事制度改革，规范中介组织从业行为。新登记各类市场主体10015户，新增注册商标48件、省级名牌产品3个。

　　县校合作行动实现新突破。新登记科研成果12项。宏源药业被授予全省知识产权示范企业，获批省级工程技术研究中心。引进实习实训大学生1364人，争取省“三区”人才32名。聘请朱英国院士等21名专家教授组成现代农业发展顾问团。

　　大别山金融工程取得新成果。金融机构创新推出扶贫贷等新产品，支持脱贫攻坚和县域发展力度不断加大。年末贷款余额67.59亿元、存款余额183.96亿元，同比分别增长16.9%和19.1%。直接融资1.66亿元，不良贷款率1.57%，两项指标位居全市第一。第七届“五个一批”活动为421家企业授信44.1亿元，落实率超过90%。城投、交投、旅投融资7.25亿元。食为天等3家企业成功在“四板”挂牌。

　　电子商务发展创造新业绩。建成并运营电子商务创业园、电子商务公共服务中心、“赶街”县级运营中心和150个村级服务站，开展电商培训2.1万人次，新增网商企业25家、网店730家。拓宽了农产品销售渠道，实现网络交易额2.8亿元、同比增长12倍。支持贫困户开办网店85家、微店78家，带动贫困人口在电商产业链中就业过1000人，增收节支过2000万元。罗田电商被商务部、省委办公厅重点推广，被人民日报、湖北日报等多家媒体聚焦关注。

　　（五）着力统筹协调，城乡面貌不断改观

　　规划体系更加完善。编制完成143个村庄规划，实现村庄规划全覆盖。编制城区9个专项规划、文化小镇规划、传统村落保护规划和天堂湖湿地公园修建性详细规划，城市设计工作获全省二等奖。圆满完成第一次全国地理国情普查工作。

　　城市建管更加精细。开展国家园林县城创建工作，新增绿地面积15万㎡。刷黑道路20万余㎡，同步完成标线划设、标牌设置、公交站场和消防栓建设。新铺设供水主管网51公里、污水主管网23公里。大别山客运中心、江夏大桥、周锡恩大道、拨云尖公园等建成投入使用。城南新区城中村改造签订协议324户、征收145户。“四大专项整治”依法追缴税费7500万元。城市管理实现常态化、规范化和精细化。

　　村镇面貌更加靓丽。整合项目资金支持村镇建设和农村生活垃圾综合治理工作。凤山、九资河、三里畈、胜利集镇和新农村建设力度加大，匡河库区、白莲石材集中区综合整治成效显著，骆驼坳燕窝塆、大河岸高庙、白庙河潘家塆、河铺林家咀、平湖粉壁坳、大崎刘家庙等示范点焕然一新。

　　生态保护更加有力。全面推进“绿满罗田”行动，完成绿化面积3.55万亩，有效防治森林病虫害。持续推进“五个禁止”，加强化工、畜牧等重点行业整治，依法关停一批项目，查处违法建设案件219起。加强集中饮用水水源地保护，“引凤入城”工程解决18万人安全饮水问题。新增光伏发电2.5兆瓦，实现清洁能源入户4682户。

　　（六）着力社会建设，民生福祉不断改善

　　社会保障全面完善。支持创业就业，发放创业扶持及贷款贴息资金1192万元，新增城镇就业5828人。新开工保障性住房3481套、建成2066套，完成农村危房改造1480户、农房抗震改造1260户。按标施保核定城乡低保17185户、27391人，提高低保、五保、优抚对象补助标准，加大重病患者、残疾人和孤儿救助力度，共发放资金1.76亿元。落实老年人、残疾人乘坐城市公交车免费优待政策。

　　社会事业全面发展。推进教育均衡发展，实施中小学校建设项目168个，发放各类助学资金3416万元；高考一本上线率、增长率均高于全省平均水平，“阳光校餐”试点工作获国务院督导组好评，教师“国培计划”经验在全国交流。新党校、行政学校建成。提高卫计服务能力，着力化解县医院建设债务，推进万密斋医院改革，新建“五化”村卫生室39家；新农合补偿参合居民100余万人次、医药费2.11亿元；发放计生奖扶资金2300万元，出生人口性别比下降5.4个单位值；成功防控人感染H7N9禽流感疫情。文化、广电、体育事业蓬勃发展，举办首届农民读书节和文化艺术节，广播“村村响”、电视“户户通”和文化广场建设工程扎实推进，罗田新闻网暨手机电视开通上线，电影《梦行者》被列为全国百部优秀国产影片，罗田健儿在市四运会上取得优异成绩。

　　社会治理全面加强。完成社区换届。深入推进“平安罗田”、“法务前沿工程”建设，着力构建社会治安立体防控体系，健全完善社会矛盾纠纷化解机制，严厉打击违法犯罪活动，严格安全生产、消防安全和食品药品安全监管，妥善处置各类突发事件，社会大局保持和谐稳定。

　　与此同时，认真做好国防动员、人民防空和民兵预备役工作，支持驻县消防、武警部队建设，关心老干部、妇女和未成年人，支持工会、共青团、妇联、文联、工商联、科协等群团组织工作，统筹推进民族、宗教、对台、外侨、保密、档案、史志、气象、保险、公积金等工作，各项工作都取得了新成绩。

　　（七）着力自身建设，行政效能不断提升

　　依法行政。认真贯彻县委决策部署，坚决执行县人大决议决定，积极转化运用县政协调研成果，自觉接受人大工作监督和法律监督、政协民主监督以及新闻媒体和社会各界监督，共办理党代表意见49件、人大代表建议55件、政协委员提案112件，回复率100%，满意率较往年显著提高。

　　规范运行。建立完善部门预算、财政资金拨付和使用管理、公务接待、村级零招待等系列制度，清理政府规范性文件21份。盘活财政存量资金4.24亿元，转换存量债务5.8亿元。评审财政投资项目287个，节约资金1.02亿元，节约率14.53%。审计项目38个，规范管理资金5.11亿元。加强政府采购管理，节约资金2140万元，节约率12.1%。“三公”经费同比削减832万元，在上年下降39.1%的基础上再下降23.1%。

　　从严管理。扎实开展“三严三实”专题教育，强化党风廉政建设主体责任，增强政治纪律、政治规矩意识。加大行政监察和执纪问责力度，立案查处210件、纪律处分193人、移送司法机关处理7人。深化专项治理，持续整治“四风”，查处工作作风问题71起、问责108人。认真做好省委巡视、专项督察、电视问政等反映问题的整改工作。

　　各位代表，2015年各项目标顺利实现，标志着全县“十二五”圆满收官。五年来，面对巨大的经济下行压力和繁重的改革发展任务，我们在县委的坚强领导下，团结依靠全县人民，求真务实，开拓进取，全县经济社会发展迈上了新台阶。

　　这五年，是综合实力全面提升、经济结构不断优化的五年。县域经济稳中有进、进中向好，综合实力在全省排名前进7位，主要经济指标增速高于全省、全市平均水平。全社会固定资产投资、规模工业企业户数、社会消费品零售总额、外贸出口、地方财政总收入、地方公共财政预算收入、农村居民收入、金融机构存款和贷款余额等9项指标实现翻番。农业结构调整力度加大，工业转型升级步伐加快，旅游、金融保险、房地产、电子商务迅猛发展，三产结构由24.6：43.4：32调整为21.2：38.5：40.3。

　　这五年，是发展战略全面完善、生态优势更加巩固的五年。立足罗田资源条件，着眼罗田长远发展，确立生态经济示范县和绿色发展先行区的发展定位，持续推进生态立县、工业强县、旅游兴县、民生和县战略，获批大别山国家自然保护区和3个国家级公园，划定了1658平方公里生态红线，绿色GDP总量和生态环境质量处于全省、全市前列，被授予国家级可持续发展实验区。

　　这五年，是城乡建设全面加快、环境面貌显著改观的五年。共争取国家项目资金51.2亿元，带动全社会固定投资605.7亿元。完成义水北路片区旧城改造，黑化主次干道80万m2，绿化120万m2，公交、公园、公厕等设施逐步完善，创建省级园林县城和卫生县城。园区扩容3000亩，引进项目60个。建成2个4A景区、2个3A景区和一批新景点，被授予全国休闲农业与乡村旅游示范县和湖北旅游强县。两次承办大别山片区扶贫开发和试验区建设现场会。交通电力通讯条件全面改善。文化小镇和美丽乡村建设亮点纷呈，三里畈、九资河、胜利被列入全国重点镇，九资河被评为全国美丽宜居小镇示范镇，官基坪、瓦屋基、潘家塆、肖家塆等村入选全国传统保护村落。

　　这五年，是社会事业全面发展、民生福祉持续改善的五年。累计投入民生工程和社会事业发展资金80.5亿元，占公共财政预算支出的66%。优先发展教育，投入资金26.3亿元，新建改扩建校舍面积20.58万㎡，发放助学资金1.79亿元，义务教育均衡发展通过国家验收。整合卫生计生资源，医疗卫生条件全面改善，新农合累计补偿医药费6.1亿元。建成保障性住房7803套、55.1万㎡，居全省前列。新建改建社会福利中心和乡镇福利院，发放社会救助和优抚补助资金6.73亿元。发放惠农直补资金3.08亿元、新农保资金2.03亿元。社会治安环境优良，荣获全国平安县和全国法治先进县称号。

　　这五年，是改革创新全面推进、对外影响不断扩大的五年。完成政府机构、行政审批、财政预算、公交体制、农村“三资”管理等重点领域改革任务，出台支持现代农业、新型工业、生态旅游、招商引资、精准扶贫、村镇建设等政策措施，创新推进金融工程、县校合作、社会治理等工作。精准扶贫干出了“罗田经验”，农村电商创造了“罗田速度”，美丽乡村形成了“罗田模式”，城建城管树立了“罗田标杆”，生态旅游书写了“罗田作为”，平安建设彰显了“罗田形象”，32项工作走在全国、全省前列，罗田知名度、美誉度显著提高。

　　各位代表，这些成绩的取得，是县委正确领导的结果，是县人大、县政协监督支持的结果，是全县人民和社会各界共同努力的结果。在此，我谨代表县人民政府，向全县广大干部群众，向各位人大代表、政协委员，向离退休老领导、老同志，向驻县部队和武警消防官兵，向所有关心、支持、参与罗田建设发展的各级领导和社会各界人士，表示衷心感谢并致以崇高敬意！

　　在看到成绩的同时，我们也清醒地认识到，全县经济社会发展还存在不少困难和问题：一是经济总量偏小，增长速度不快，质量效益不高，结构调整任务艰巨；二是贫困人口多，增收渠道窄，县乡村收入来源不足，脱贫摘帽任务十分繁重；三是城镇化水平不高，城乡发展不平衡，公共基础设施建设相对滞后，公共服务能力不足；四是改革任务重，创新能力弱，人才队伍相对匮乏；五是少数政府工作人员存在政治规矩、政治纪律意识不强、法治观念淡漠和庸政、懒政、怠政等问题。对此，我们必须增强忧患意识、责任意识和担当意识，努力在转型升级、提质增效、补齐短板、优化服务上实现新突破。

　　二、“十三五”时期的指导思想、目标和要求

　　“十三五”时期，是罗田实现精准脱贫、同步小康的决胜期，是经济转型、后发赶超的机遇期。根据《中共罗田县委关于制定罗田县经济和社会发展第十三个五年规划的建议》，县政府深入调查研究并广泛听取意见，认真编制了《罗田县经济和社会发展第十三个五年规划纲要（草案）》，提请大会审议。

　　“十三五”时期经济社会发展的指导思想是：高举中国特色社会主义伟大旗帜，全面贯彻落实党的十八大、十八届三中、四中、五中全会精神，以马克思列宁主义、毛泽东思想、邓小平理论、“三个代表”重要思想、科学发展观为指导，深入贯彻习近平总书记系列重要讲话精神，围绕“五位一体”总体布局和“四个全面”战略布局，坚持“五大发展理念”和“三维纲要”，突出“双强双兴”和“四大行动”[5]，推进生态立县、农业稳县、工业强县、旅游兴县、民生和县发展战略，把罗田建设成为精准脱贫样板区、生态保护与建设示范区、绿色发展先行区。

　　今后五年全县经济社会发展的总体目标是：经济保持平稳较快发展。主要经济指标增速力争高于全市平均水平，发展质效明显提升。地区生产总值年均增长9%左右，地方公共财政预算收入年均增长12%左右，综合实力进入全省三类县市前列。人民生活水平显著改善。城乡居民人均可支配收入年均增长10%左右，常住人口城镇化率达到50%，现行标准下农村贫困人口全部脱贫，贫困村全部出列，贫困县摘帽。社会文明程度大幅提升。社会主义核心价值观和中华民族传统美德深入人心，公民文明素质全面增强，以信用信息资源共享为基础的全社会征信系统基本建成。生态环境质量更加优良。水、大气、土壤污染等环境问题得到有效遏制，主体功能区规划全面实施，水土保持型生态功能区建设初现成效，全县森林覆盖率力争提高到72%。社会治理水平全面提高。社会主义法治精神得到大力弘扬，依法治县工作深入推进，党委领导、政府主导、社会协同、公众参与、法治保障的社会治理体制全面完善，社会治安“五率”等指标保持在全省、全市先进行列。创建国家园林县城、国家卫生县城、国家生态县、国家旅游标准化示范县和省级文明县城，夺取全国社会治安综合治理“长安杯”。

　　为实现上述目标，应着重把握好以下五个方面：

　　（一）推进创新发展，培育增长新动力。把创新作为引领发展的第一动力，实施创新驱动发展战略。激发改革创新活力，加快供给侧结构性改革，落实去产能、去库存、去杠杆、降成本、补短板五大任务。培育创新产业体系，稳步发展现代农业，实施《中国制造2025》[6]，加快发展休闲旅游、养生养老、金融保险等现代服务业，推进“互联网+”行动计划，促进一二三产业协同融合发展。创新投融资机制，深入实施“大别山金融工程”，对接武汉城市圈科技金融改革，健全财政资金引导配置机制、多方协同融资服务机制、金融生态建设长效机制。深化县校合作，培育尊重知识、崇尚创业、追求卓越的创新文化，推动大众创业、万众创新。

　　（二）推进协调发展，形成发展新格局。坚持区域协同、城乡一体、经济社会协调、物质文明精神文明并重，统筹县城、集镇、村庄建设，构建“一心、四柱、三画廊”城乡一体化格局。坚持“三个转变”[7]，强化“四精”[8]理念，建设宜居宜业宜游的山水园林城市、海绵城市[9]、智慧城市。加快特色集镇、文化小镇、美丽乡村和交通、电力、通信等基础设施建设，兼顾重点镇与小城镇、贫困村与一般村建设。放开户籍限制，支持农村居民进城、进镇、进中心村和居民点，实现基本公共服务常住人口全覆盖。落实全面二孩政策，综合治理出生人口性别比。加快人防工程建设，促进军民融合发展。深入开展文明创建活动。

　　（三）推进绿色发展，建设美丽新家园。坚持“绿水青山就是金山银山”的发展理念。发展绿色产业，推进产业生态化、生态产业化，促进资源优势加快转化为经济优势。优化空间布局，推行“多规合一”，分片统一建筑风格。加强环境保护和治理工作，实施最严格的环境保护、水资源管理、节约用地和耕地保护制度，落实主体功能区规划，综合防治水土流失、地质灾害和土壤、河流、大气污染。开展绿满罗田、清洁家园行动，巩固“五禁”成果，加大工业、旅游、畜牧水产养殖、石材开采加工等重点行业治理力度，加快垃圾、污水处理设施和重点节能减排项目建设步伐。改革环保执法体制，实行领导干部自然资源资产离任审计、生态环境损害赔偿和责任追究制度。

　　（四）推进开放发展，开创共赢新局面。积极融入长江经济带、中部城市群、武汉城市圈和大别山生态经济带建设，在更高视角、更大范围优化资源配置，更好发挥罗田比较优势和后发优势。围绕大别山连片特困地区脱贫攻坚、试验区“十年大跨越”目标，推进大别山区域合作，加快交通基础设施互联互通，促进优势资源和生产要素互惠互动，探索建立大别山旅游、特色产业、碳汇交易协作区，创建大别山世界地质公园，共同打造乡村生态文化和休闲度假旅游目的地。优化开放发展环境，建立完善清单管理制度，吸引更多县外资金、人才和技术。

　　（五）推进共享发展，实现民生新改善。按照人人参与、人人尽力、人人享有要求，坚守底线、突出重点、完善制度，加大财政投入，着力补齐短板，增加公共服务供给，提高群众获得感、幸福感。实施脱贫攻坚工程，确保提前全面精准脱贫，与全国同步建成小康社会。坚持就业优先，实现更高质量就业。完善社会保障体系，实施全民参保计划。推进救助制度整合，发展社会福利和慈善事业，关爱农村留守儿童、妇女和老人。加强保障性住房建设管理。办好人民满意教育，优化学校布局，抓好学前教育，推进义务教育优质均衡发展，普及高中阶段教育，加强现代职业教育，构建继续教育、终身教育体系。加快卫生计生事业改革发展，深化公立医院改革，发展社会办医，完善分级诊疗，建立统一的城乡居民医保制度，构建维护公益性、调动积极性、保障可持续性的医疗卫生服务体系。建立基本公共文化服务体系、产业体系、市场体系。加强食品药品、安全生产等公共安全管理。坚持用法治思维和法治方式推进社会治理，维护公平正义，促进社会和谐。

　　三、2016年主要工作

　　2016年是“十三五”的开局年，是圆满完成届期目标的决战年。做好今年的政府工作，我们必须把思想和行动统一到中央、省、市委政府和县委对形势的科学判断上来，深化对我县经济社会发展阶段性特征的认识，把握“十三五”规划、大别山革命老区振兴发展规划、大别山革命老区经济社会发展试验区规划实施的战略机遇，着力推进供给侧结构性改革和精准扶贫工作，努力实现经济社会又好又快发展。

　　今年的主要预期目标是：地区生产总值增长9%，规模工业增加值增长15%，全社会固定资产投资增长17%，社会消费品零售总额增长13%，外贸出口增长10%，地方公共财政预算收入增长15%，城乡居民人均可支配收入增长10%，居民消费价格涨幅控制在3%以内，城镇登记失业率控制在4%以内。全面完成年度脱贫任务和省市下达的安全生产、节能减排、耕地保护等责任目标。

　　实现上述目标，我们将重点抓好以下八个方面的工作：

　　（一）坚持精准扶贫，确保精准脱贫

　　突出产业精准扶贫。开展“强基础、兴产业、促脱贫”为主题的第六轮“三万”活动，采取“五位一体”[10]等多种模式，因地制宜、因户施策培育和兴办脱贫致富产业。重点发展黑山羊、中药材、板栗、旅游、电子商务等五大产业，统筹扶持发展光伏发电等其他扶贫产业。支持市场主体带动贫困户脱贫，给予产业发展调度资金、贷款贴息和技术服务等支持。探索建立生态补偿机制，扶持国有林场村、库区村转型发展，壮大村级集体经济。

　　加快易地搬迁扶贫。采取“五统一”、“四位一体”、“七子”工作法[11]、集中与分散结合方式，帮助引导贫困户向中心村、移民新村、小城镇等集中安置点搬迁，确保搬得出、稳得住、能发展、可致富。有意愿同步搬迁到集中安置点的一般农户可享受公共基础设施配套。鼓励贫困户进城进镇购买商品房并给予购房补贴。成立县易地扶贫搬迁投融资平台，加大融资和项目整合力度，支持集中安置点和贫困村“十通十有”[12]建设。

　　加强政策兜底扶贫。医疗救助一批，采取城乡居民医疗保险、大病补充保险、大病医疗救助和社会救助等措施，实现因病致贫群众救助全覆盖。教育助学一批，整合各类助学资源，按一个口径、“三零”[13]目标, 实现建档立卡贫困户子女就学资助全覆盖。培训就业一批，对有劳动能力的贫困人口分类进行技能培训，实现贫困劳动力就业培训全覆盖。“两无”兜底一批，对无劳动能力、无脱贫能力的贫困户，通过农村低保、五保、孤儿和残疾人救助等途径进行救助，实现政策性兜底全覆盖。争创全省精准扶贫工作先进县。

　　（二）坚持多业并举，加快转型升级

　　发展现代农业。稳定粮油生产，加快农田水利建设，推进农业三项补贴[14]合并为农业支持保护补贴改革，建设5万亩优质粮油生产基地。推进农业适度规模经营，采取土地流转、托管、入股等方式，完善利益联结机制，支持龙头企业和新型农村经营主体发展。推进农业标准化生产和品牌化营销，建设黑山羊、黄牛、肉鸡等良种繁育基地，板栗、甜柿等特色经济林基地，中药材GAP种植基地，食用菌、有机鱼等特色种养基地。发展休闲观光农业和“智慧农业”，培育家庭农场和农村电商示范户。推进国有林场和供销合作社改革，基本完成农村土地承包经营权确权登记颁证和第三次全国农业普查工作。

　　壮大新型工业。坚持围绕财源抓工业、围绕园区建平台、围绕企业强产业。优先安排项目资金、用地、水电等要素，重点培育产业龙头企业、创税创汇大户、就业带动型和科技成长型企业。实施园区扩容提质工程，启动2000亩土地征收、骆驼坳许家冲村搬迁和三赵线[15]建设工作，推进纺织产业园等项目建设，新建成投产项目15个。依法全面清理项目闲置用地。加快小微企业园建设。严把石材产业园项目评审关，做好尾矿综合治理和利用工作。深化企业服务行动，及时落实结构性减税、出口退税、电价调整等优惠政策，为企业发展减负松绑。做好困难企业解困和企业改制收尾工作。全年新增规模企业20家，实现规模工业增加值30亿元。

　　推进全域旅游。加快核心景区建设，以天堂寨、薄刀峰、大别川百里生态画廊为主体，创建罗田大别山国家5A级景区。打通天堂寨景区外围交通路网，改造升级千金街外环线等三条旅游公路，畅通天堂寨景区内部游线微循环，规划建设游客集散中心和停车场，启动天堂古寨、主峰索道等建设。推进大别川百里生态画廊6个节点项目建设和杆线整治工作。支持三里畈温泉度假区、胜利老街和烈士陵园创建4A级景区，天堂湖创建3A级景区。开展“旅游质量提升年”活动，推进旅游标准化和“厕所革命”，规范“农家乐”经营行为。加大旅游营销，开拓华东、华南客源市场，做好节假日错峰调度工作。理顺旅游管理体制，实现管理规范化、常态化。筹建大别山旅游集团，力争在“新三板”上市。争创全省生态旅游示范县和国家全域旅游示范区。

　　培育新兴产业。加快发展网络经济，完善电子商务应用平台，推动互联网与农业、工业、旅游及商贸服务业融合发展，改变传统营销模式，促进线上线下互动，创建全国电子商务进农村综合示范县。规范物流快递行业发展，适时启动物流园建设。大力发展文化创意、养生养老等现代服务业。

　　（三）坚持项目拉动，夯实发展基础

　　大干项目建设。开展“重大规划项目启动年”活动。加强政策研究，用好脱贫攻坚“金字招牌”，做好项目策划和对接入库工作。重点谋划推进沿江高铁罗田段、大别山红色高速公路等10个重大基础设施项目和平坦原抽水蓄能、风力发电等10个重大产业项目，做好项目前期工作，力争早日获批投资计划。加快项目建设，实行项目“六制”管理[16]，制定作战图、路线图和时间表。加大督导协调和考核奖惩力度，确保全年争取国家项目资金12亿元以上。

　　深化招商引资。明确招商方向，着力引进清洁能源、纺织服装、农特产品深加工、文化旅游、电子商务、现代服务业等低排放、高税收项目。创新招商模式，坚持全员招商与专班招商、定向招商与产业招商、招商与安商结合，紧盯武汉城市圈、长三角、珠三角等重点地区，做好项目策划推介工作，精准对接产业转移、协作配套。确保全年引进新开工重点项目20个，到位资金10亿元。

　　推动消费升级。实施质量兴县战略，完善标准体系，加强质量监管，支持企业品牌建设，提高供给效率。优化消费环境，发展消费信贷，加强社会信用体系建设，规范市场秩序，健全消费者权益保护机制。完善农村商品市场网络，推动农村流通网络升级。扩大信息消费，消除移动通信信号盲区，提高农村光纤网络覆盖率。落实房地产去库存各项政策措施，扩大保障性住房货币化安置比例，支持房地产企业参与易地扶贫搬迁工作。

　　（四）坚持改革创新，激发发展动能

　　推进市场主体增量提质工程。加大简政放权力度，做到“放、管、服”三管齐下，建立网上申报、并联审批、在线服务运行机制，规范中介组织发展，加大政府购买服务力度，促进大众创业、万众创新，力争新增市场主体5000家以上。

　　推进县校合作工程。加强科研院所常态化合作，共建校企研发中心，以协同创新带动自主创新，促进科技成果转化，突破中药颗粒剂GMP生产、板栗等特色农产品深加工关键技术。开展实用技术培训，大力培育乡土人才。抓好“双实双业”[17]工作，引进实习实训大学生1500名、创业就业500名。

　　推进大别山金融工程。引进金融投资主体，加强与省市各种基金对接，做大做强担保公司、村镇银行、小贷公司，提高城投、园投、旅投、交投融资能力，支持企业市场化兼并重组和上市融资。创新政银保企合作方式，县财政设立1.2亿元县级调度资金和1亿元过桥资金[18]，着力缓解企业融资难、融资贵问题。优化金融生态环境，完善诚信激励和失信惩戒机制，办好全省农村金融改革试点，争创全省最佳金融信用县和全省保险先进县。

　　（五）坚持以人为本，促进城乡协调

　　完善城市功能。重点推进“一带一桥、一洞五路、五园五场、五大工程”建设。打造滨河风光带，做好江夏大桥、界河大桥两端生态护岸、亲水平台、景观节点建设工作；维修加固东门巴源大桥；打通北城岗隧道及周边区域五条道路；建设拨云尖生态公园、老塔山森林公园、党家山山地公园、北城岗休闲公园、大士阁山谷公园的主入口、主园路和主要节点，完善市民广场、体育场、停车场、菜市场、垃圾处理场等设施；加快旧城改造、路网畅通、安居保障、绿化升级、人武人防等配套工程建设。

　　加快村镇建设。按照“一年起步、三年见效”目标，统筹生态村、美丽乡村、文明新村、新农村示范村建设以及传统村落保护、易地扶贫搬迁等工作，整合各类项目资金，重点支持50个县级示范村、20个市级试点村和10个精品村建设。改造松子关至河铺、河铺至大地坳等公路。

　　加强城乡管理。借助“互联网+”，打造现代化城市治理模式。完善社区规范化建设，提高网格化管理效率。加强城区交通秩序管理，做好重点时段、重点区域疏堵保畅工作。加大城乡规划实施力度，建立部门、乡镇、村组联动机制，坚决遏制违规违法建设行为。

　　（六）坚持生态优先，推进绿色发展

　　加强环境保护。实施“蓝天工程”，落实秸秆禁烧和城区禁鞭措施，推进燃煤锅炉和重点行业除尘改造，加强工业废气、建筑扬尘、餐饮业油烟治理。实施“绿地工程”，启动大别山南麓天然林保护工程，持续推进“绿满罗田”和禁伐行动，抓好村庄河堤通道绿化、森林防火、红叶景观林保护工作。实施“碧水工程”，加强规模化畜禽养殖污染防治、集中式饮用水水源地保护、天堂湖湿地修复、白莲河库区综合开发等工作，启动重点集镇污水集中处理设施建设。实施“净土工程”，推进土壤污染防治和修复工作，深入开展城乡生活垃圾治理及环境综合整治行动，实现治理工作常态化。

　　促进产业转型。实施重点行业清洁生产提升计划和循环经济发展行动方案，建立覆盖所有固定污染源企业排放许可制度，探索差别地价等措施，促进清洁生产、绿色发展。开展国家生态县、国家绿色能源示范县、国家绿色旅游示范基地创建活动。

　　强化考核问责。严格落实新《环保法》和《湖北省土壤污染防治条例》，实行最严格的环境保护制度，强化节能减排目标考核。严格环境执法，实行环境问题约谈、问责、追责。

　　（七）坚持共建共享，保障改善民生

　　做好促进就业工作。开展“春风行动”，落实扶持政策，促进高校毕业生、农村转移劳动力、退役军人创业就业，做好企业下岗职工、失业人员再就业工作，鼓励支持返乡创业，新增城镇就业6500人，支持1000人创业。

　　完善社会保障体系。深化社保、医保、户籍等制度改革。完成全民参保登记，促进被征地农民、城镇灵活就业人员参保，全面落实机关事业单位养老保险制度改革。构建“大救助”机制，衔接各类医疗、保险、救助制度，整合城乡居民基本医疗保险制度，全面实施城乡居民大病保险并纳入即时结算系统。推进低保按标施保工作常态化，加大重大疾病患者、重性精神病人、重度残疾人的救治救助力度，做好农村福利院日常管理工作，落实困难残疾人补贴制度。

　　加快社会事业发展。优先教育发展。健全定期议教和校园安全工作联席会议制度。优化城乡教育资源布局，启动县幼儿园迁建和新建一所高中的前期准备工作。探索建立农村留守儿童关爱帮助机制和社会体系，开展“圆梦助学”等系列帮扶活动。弘扬孝德文化，关心关爱留守老人和妇女。推进医疗卫生与养老服务结合，采取特许经营、公建民营、民办公助等模式加快发展养生养老服务业，启动万密斋养生养老康复中心建设。实施农村医生特岗计划，加强基层执业医师队伍建设。做好全面二孩政策落地工作，提高计划生育精细化服务管理水平，综合治理出生人口性别比。加快地面无线数字电视工程、农村数字影院试点工程、村级“五个一”[19]标准化文化广场建设，举办农民读书节、“挺进大别山”自行车公路赛等系列赛事活动，开展官基坪村等古民居修缮保护。启动乡镇志编纂工作。

　　创新社会综合治理。加强法治宣传教育，启动“七五”普法工作。完善村（社区）民主自治，推进社会治理社区化、单元化、网格化和志愿者服务常态化。严格落实安全生产责任制，建立安全生产网格化管理体系，强化预防治本，实行监管区域全覆盖。加强食品药品安全监管，建设食品药品公共安全检验检测中心。深化平安创建，完善立体化社会治安防控体系，提高人防、物防、技防水平，加强网络安全和网吧管理，推进法务前沿工程、阳光信访和法律援助工作。支持群团协会组织发挥作用，统筹做好民族宗教、外事侨务、国防动员、拥军优属等各项工作。

　　（八）坚持依法行政，建设人民满意政府

　　建设法治政府。贯彻《法治政府建设实施纲要（2015—2020）》。坚持宪法法律至上、法律面前人人平等、权由法定、权依法使等基本法治理念，始终在法治轨道上做好政府各项工作。坚持依法决策、科学决策，严格遵循重大行政决策法定程序，完善政府法律顾问制度和重大决策合法性审查机制。坚持行政复议县长审案制度，完善行政执法程序和行政行为纠错机制。落实普法执法责任制，引导群众依法行使权利、表达诉求、解决纠纷，全力维护公共利益、群众权益和社会秩序。

　　建设为民政府。强化宗旨意识，深入践行群众路线和“三严三实”要求，持之以恒纠正“四风”，推进政府工作重心下移、力量下沉，主动问政于民、问需于民、问计于民，努力为人民群众办实事、谋福祉。加强公务员政策法规、专业知识培训学习，做好政策宣传解读和贯彻落实工作。

　　建设高效政府。创新政务服务，加快“智慧政务”建设，打造“办事不求人”的“网上政府”。精简优化办事程序，不断提高办事效率，实行清单化、台账式管理，推行岗位责任制、首问负责制、服务承诺制、限时办结制、责任追究制，加大督办督查、治庸问责力度，确保各项政策、决策、规划、部署快速精准落地。建立合理的“容错”机制和财政供给保障机制，鼓励大胆干事、竞进作为。

　　建设廉洁政府。严格落实党风廉政建设主体责任和“一岗双责”，深入推进党风廉政建设和反腐败斗争。坚持把纪律规矩挺在前面，认真贯彻落实《准则》和《条例》，抓好巡视反馈问题的整改落实工作，自觉养成在监督加强条件下开展工作的良好习惯。加强权力监督，完善工程建设等重点领域预防腐败长效机制。加强政务公开，实行财政预决算、“三公”经费和审计报告公开。加强审计监督，加大对审计发现问题的整改和责任追究力度。加强行政监察，严厉整治和查处损害群众切身利益的不正之风和腐败问题。加强廉政文化建设，营造清廉为官、事业有为的干事创业氛围。

　　各位代表，县政府今年继续以贴近民生需求、惠及大多数群众为出发点，整合项目资金，安排公共财力，重点办好以下“十件实事”：

　　1、实现农村建档立卡贫困户6600户、22000名贫困人口精准脱贫。

　　2、完成100公里通村通组公路和300公里农村公路安保工程建设任务。

　　3、着力解决农村供电卡口和低电压问题，投入农网改造资金2.65亿元，建成薄刀峰35千伏变电站，新建（改造）10千伏线路290公里、低压线路710公里，新增农村配变418台。

　　4、完成最后84个村的“宽带乡村”工程建设，实现所有行政村宽带网络全覆盖。

　　5、完善城乡立体化社会治安防控体系，改造一期500个视频探头，新建三期150个A类高清视频探头和8个治安卡口。

　　6、建成1055套保障性住房。

　　7、改扩建城区河东街小学和栗子坳小学。

　　8、完成城区一河两岸2公里生态护坡、6座大桥亮化工程建设，建成城区生活垃圾资源化无害化处理场、6个垃圾中转站和6个景点式公厕。

　　9、建成义水北路等地下停车场，城区新增停车泊位1000个。

　　10、完成体育中心项目征地、规划设计、工程招投标等前期工作并动工建设。

　　各位代表，新起点孕育新希望，新希望绘就新蓝图，新蓝图期待新作为。让我们在县委的坚强领导下，以奋发有为的精神状态、攻坚克难的拼搏意志、只争朝夕的紧迫劲头，锐意进取，扎实工作，为全面开创罗田绿色崛起、跨越发展新局面而努力奋斗！
